# Telestration with augmented reality improves the performance of the first ten ex vivo porcine laparoscopic cholecystectomies: a randomized controlled study

**DOI:** 10.1007/s00464-023-10360-y

**Published:** 2023-08-23

**Authors:** Amila Cizmic, Felix Müller, Philipp A. Wise, Frida Häberle, Felix Gabel, Karl-Friedrich Kowalewski, Vasile Bintintan, Beat P. Müller-Stich, Felix Nickel

**Affiliations:** 1 Department of General, Visceral and Thoracic Surgery, University Medical Center Hamburg-Eppendorf, Martinistraße 52, 20251 Hamburg, Germany; 2 Department of General, Visceral, and Transplantation Surgery, Heidelberg University Hospital, Im Neuenheimer Feld 420, 69120 Heidelberg, Germany; 3 Department of Urology, University Medical Center Mannheim, Heidelberg University, Theodor-Kutzer-Ufer 1-3, 68167 Mannheim, Germany; 4 Department of Surgery, University of Medicine and Pharmacy, Cluj Napoca, Romania; 5 Clarunis – University Center for Gastrointestinal and Liver Diseases, St. Claraspital AG, Kleinriehenstrasse 30, 4058 Basel, Switzerland

**Keywords:** Telestration, Augmented reality, Artificial intelligence, Minimally invasive surgery, Training, Cholecystectomy

## Abstract

**Introduction:**

The learning curve in minimally invasive surgery (MIS) is steep compared to open surgery. One of the reasons is that training in the operating room in MIS is mainly limited to verbal instructions. The iSurgeon telestration device with augmented reality (AR) enables visual instructions, guidance, and feedback during MIS. This study aims to compare the effects of the iSurgeon on the training of novices performing repeated laparoscopic cholecystectomy (LC) on a porcine liver compared to traditional verbal instruction methods.

**Methods:**

Forty medical students were randomized into the iSurgeon and the control group. The iSurgeon group performed 10 LCs receiving interactive visual guidance. The control group performed 10 LCs receiving conventional verbal guidance. The performance assessment using Objective Structured Assessments of Technical Skills (OSATS) and Global Operative Assessment of Laparoscopic Skills (GOALS) scores, the total operating time, and complications were compared between the two groups.

**Results:**

The iSurgeon group performed LCs significantly better (global GOALS 17.3 ± 2.6 vs. 16 ± 2.6, *p* ≤ 0.001, LC specific GOALS 7 ± 2 vs. 5.9 ± 2.1, *p* ≤ 0.001, global OSATS 25.3 ± 4.3 vs. 23.5 ± 3.9, *p* ≤ 0.001, LC specific OSATS scores 50.8 ± 11.1 vs. 41.2 ± 9.4, *p* ≤ 0.001) compared to the control group. The iSurgeon group had significantly fewer intraoperative complications in total (2.7 ± 2.0 vs. 3.6 ± 2.0, *p* ≤ 0.001) than the control group. There was no difference in operating time (79.6 ± 25.7 vs. 84.5 ± 33.2 min, *p* = 0.087).

**Conclusion:**

Visual guidance using the telestration device with AR, iSurgeon, improves performance and lowers the complication rates in LCs in novices compared to conventional verbal expert guidance.

**Supplementary Information:**

The online version contains supplementary material available at 10.1007/s00464-023-10360-y.

Minimally invasive surgery (MIS) has brought significant benefits to patients undergoing surgical treatment [1]. MIS has certain advantages compared to open surgery, such as shorter hospital stays, reduced blood loss, less tissue trauma, and faster recovery [1–3]. However, there are some challenges in acquiring MIS skills compared to open surgery.

On the contrary to open surgery, the MIS learning curve is often hindered by the fact that instructions can only be given verbally due to distance, ergonomics, and sterility of the operating room (OR) [4–6]. Intraoperative guidance and training regarding instructions, identification of target structures, and the correct use of instruments are given almost exclusively verbally or through gesturing away from the operating screen, which makes this communication prone to error [7].

Today, MIS training can be performed outside the OR using virtual reality (VR) simulators and box trainers [8–10]. The difficulties in MIS skill transfer can lead to longer operation times, increased levels of stress for the trainees, and potential risks of intraoperative complications [12]. Therefore, efficient MIS training and skill transfer can be arduous and demanding [13].

Using a telestration model with augmented reality (AR) has shown benefits in surgical performance in medical students without MIS experience [6, 14]. The iSurgeon is a telestration device with AR that allows interactive visual guidance during MIS training. It enables the tutor to visually guide the trainee by capturing the tutor’s hand and projecting it on the operating screen during MIS, identifying critical anatomical structures, suggesting instrument handling, and helping avoid common intraoperative pitfalls. The iSurgeon does not require the tutor to touch the screen and, therefore, can be used in the OR environment. It also allows remote access, so the tutor does not have to be in the same room as the trainee. However, no study reported on the efficiency of the iSurgeon in trainees with previous basic MIS experience performing a standard MIS procedure such as laparoscopic cholecystectomy (LC) repetitively.

The study's primary aim was to assess whether the iSurgeon has positive learning effects on MIS training in trainees with basic MIS experience performing repetitive ex vivo porcine LC compared to conventional verbal instructions.

## Materials and methods

### Study design and participants

This study was designed as a randomized, controlled two-arm study. The total sample size was 40 trainees. The study was performed as a part of the complementary MIS training curriculum for medical students (trainees) in their clinical years (3rd to 6th year) at Heidelberg University Medical School, Germany. The training was conducted in the MIS training center within the Department for General, Visceral, and Transplantation Surgery, University Hospital Heidelberg, Germany. The study was approved by the local ethics committee (S-436/2018).

**Fig. 1:**
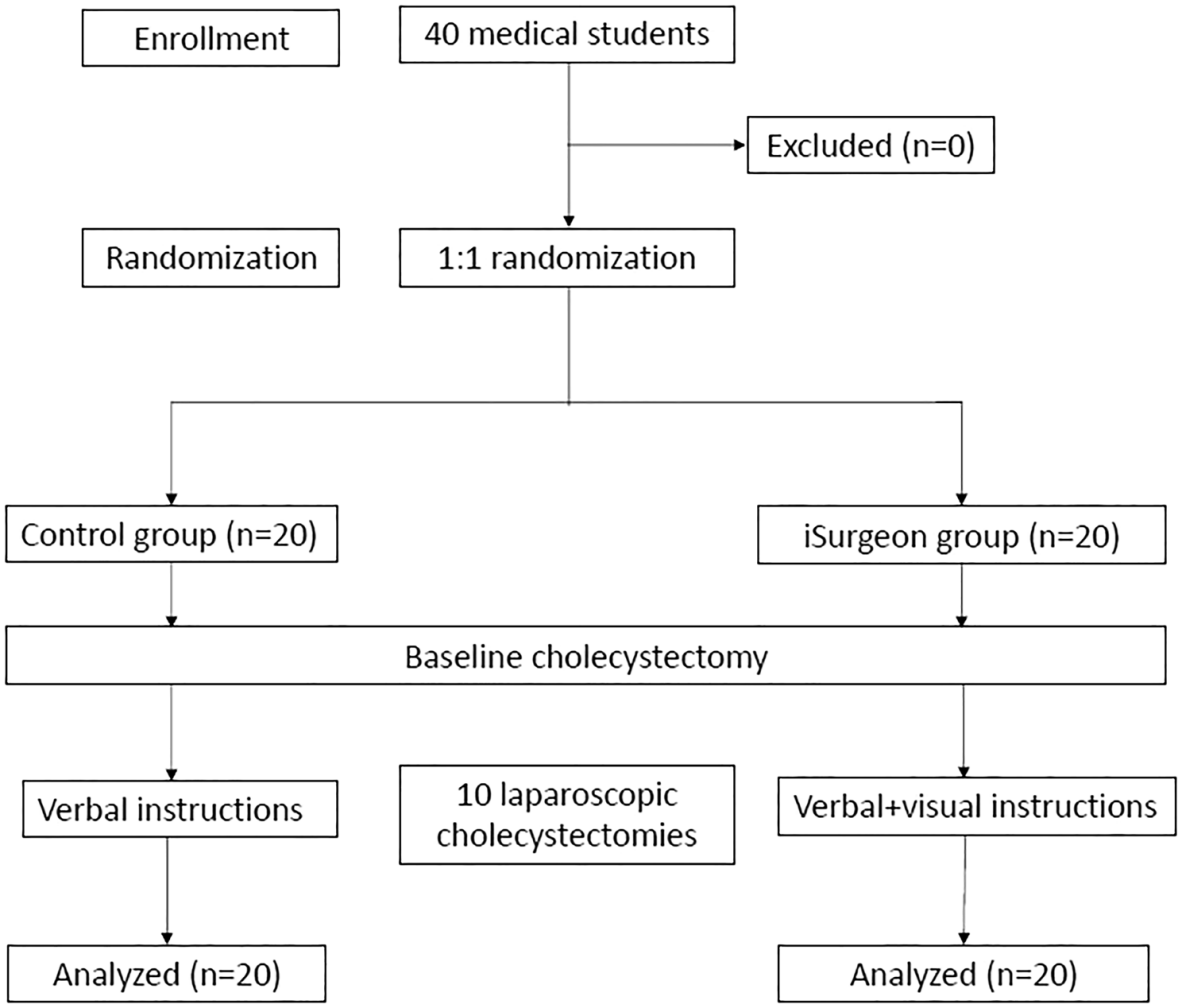
Flowchart of the randomized controlled study

After signing a consent form, the trainees were randomized into two groups. Randomization was performed by an independent employee, otherwise not involved in planning, conducting, or analyzing the study, using a free available randomization tool (https://ctrandomization.cancer.gov/tool/). After the randomization of 40 trainees into the iSurgeon group (n = 20) and control group (n = 20), every trainee performed a baseline LC during which both groups only received verbal assistance upon request (Fig. 1).

**Fig. 2:**
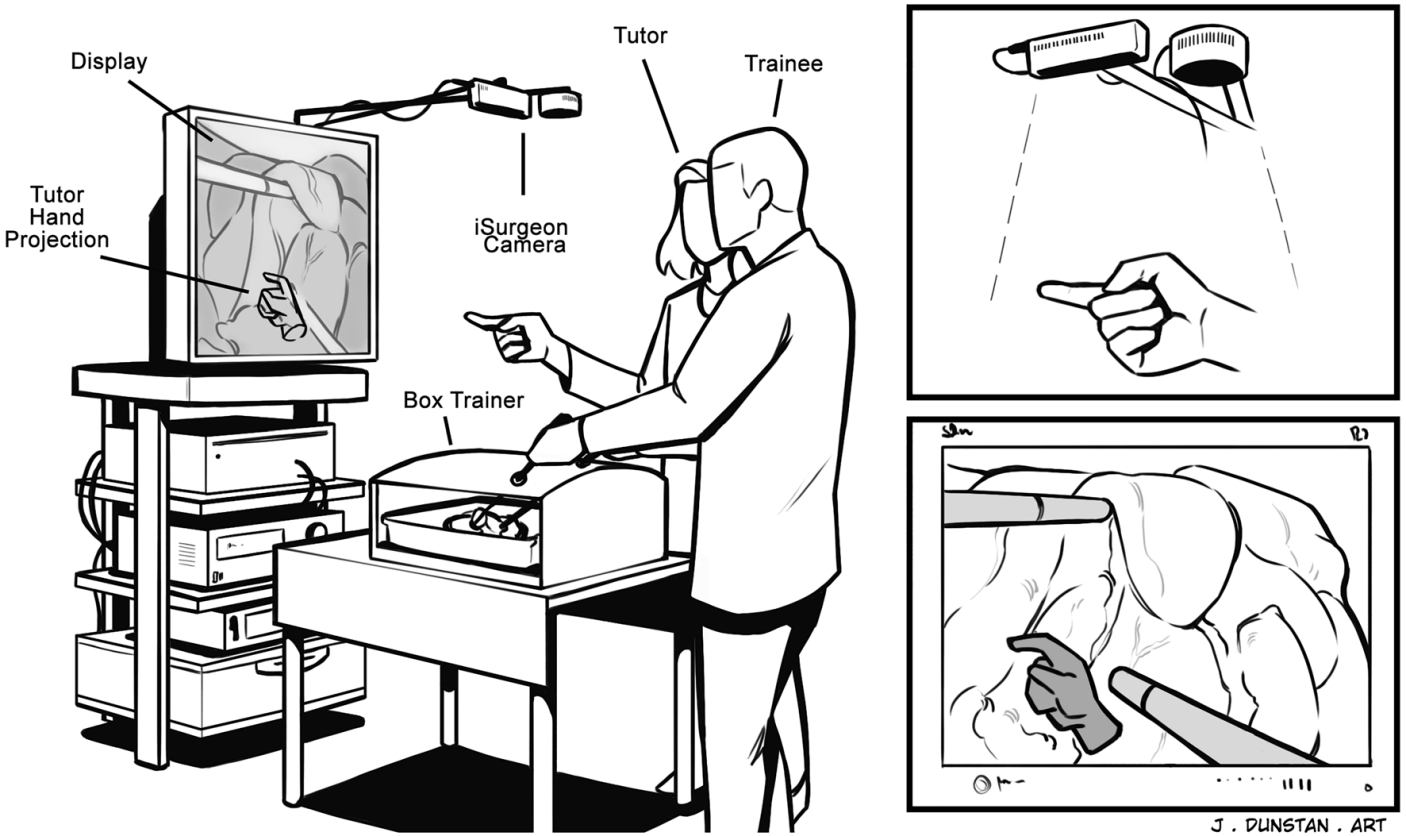
iSurgeon setup during a LC on a box-trainer

**Fig. 3:**
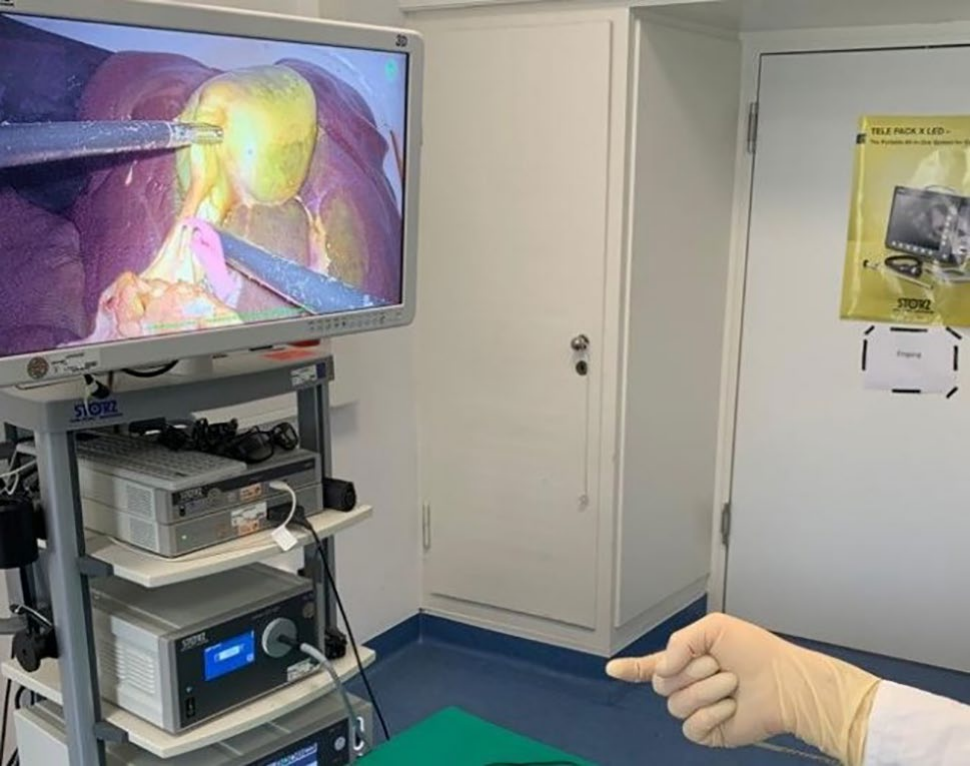
Tutor giving instructions during ex vivo porcine LC to the medical student with hand gestures displayed on the screen

After performing the baseline LC, each trainee performed 10 LCs within ten training sessions. The trainees within the control group only received verbal guidance, whereas those within the iSurgeon group received verbal and visual guidance using the iSurgeon (Fig. 2). The visual guidance was provided by a trained tutor, whose hand gestures were projected on the laparoscopic screen in real-time using the iSurgeon (Fig. 3). The training sessions were conducted by a tutor specifically trained in MIS and LCs on ex vivo porcine livers.

## Materials

The study was performed on a Szabo–Berci–Sackier Box Trainer and a standard laparoscopy tower (KARL STORZ GmbH & Co. KG, Tuttlingen, Germany). A study-specific task station was specifically constructed for this study. The iSurgeon was developed at the Department of General, Visceral, and Transplantation Surgery at Heidelberg University Hospital within a federally funded EXIST program and was provided for this study. For the performed LCs, a fenestrated gall bladder grasper, curved scissors, clip applicators, and laparoscopic monopolar hook electrode were used (KARL STORZ GmbH & Co. KG, Tuttlingen, Germany).

In this study, the training was conducted by a medical doctor who had reached proficiency by performing at least 30 ex vivo porcine LCs himself. Furthermore, the tutor was monitored by a board-certified surgeon. Evaluation of both groups was done by one and the same tutor to avoid potential bias when evaluating the student’s performance. The tutor was also trained on how to use the iSurgeon and gave instructions on it. Evaluation of the complications and difficulty of the LCs was directly done at the end of the training to review the condition of the used organs. Furthermore, all training sessions were captured on video recordings for later performance assessments by a blinded rater.

### The iSurgeon

The iSurgeon is a collaborative telestration device with AR that enables interactive visual guidance during MIS procedures. With a specifically designed camera, the iSurgeon captures the tutor’s hand above the surgical field and projecting it on the operating screen. It allows tutors to draw, annotate, and provide the identification of the anatomical structures, suggests instrument handling, and helps to limit mistakes. The trainee can see the tutor’s hand and/or annotations overlaid on the surgical field on the operating screen, suggesting the next step in the MIS procedure or the plain of dissection. The iSurgeon also allows remote access, so the tutor does not have to be in the same room as the trainee. This real-time communication allows for instant feedback and helps enhance decision-making and surgical precision.

### Laparoscopic cholecystectomy on an ex vivo porcine liver

After inserting the instruments and identifying critical anatomical structures, the gallbladder's neck was grasped with consequent luxation and identification of Calot's triangle (trigonum cholecystohepaticum) [15]. From this point, both groups received verbal assistance on-demand or if they experienced difficulties during the following operation steps. On-demand verbal guidance was provided if the trainees expressed need for help/guidance during an LC or the trained tutor assessed the need to guide the trainees so they could proceed with the surgical procedure. The iSurgeon was used to assist the iSurgeon group in addition to verbal guidance throughout the LC procedure.

The next step was the preparation of Calot's triangle blunt or with diathermy. Fibrous tissue strands could be severed under sight only. Assistance with the iSurgeon on how to and where to best start coagulating was provided. The iSurgeon system assisted in identifying the cystic duct and artery and achieving the critical view of safety (CVS). CVS is seen as the gold standard to reduce the risk of bile duct injury in LC and was defined by Strasberg in 1995 as clearance of the hepatocystic triangle of fat and fibrous tissue, exposure of the cystic plate, and two and only two structures to be seen entering the gallbladder [16]. Subsequently, clipping and cutting through the cystic artery and duct were performed. Trainees applied two clips proximally and one clip distally, with a spacing of approximately 0.5 cm between the clips. Once more, visual guidance with iSurgeon was provided while applying clips and cutting the anatomical structures. Next followed the detachment of the gallbladder in a retrograde manner where the gallbladder was grasped at the neck, carefully luxated cranially, and step by step detached from the biliary fossa. Assistance in holding the gallbladder and identifying and visualizing the proper anatomical layers and possible coagulable structures to prevent damage to the gallbladder and biliary fossa were shown using the iSurgeon in the iSurgeon group. Upon final gallbladder detachment, a final assessment of the operating field for major damage was done, marking the end of the procedure.

### Outcome parameters

The primary outcome of the study was performance scores assessed using standardized Objective Structured Assessments of Technical Skills (OSATS) (task-specific and global) and Global Operative Assessment of Laparoscopic Skills (GOALS) (task-specific and global) scores for all LCs performed. Global and task-specific OSATS and GOALS scores are considered reliable for assessing MIS training [17–19].

Secondary outcomes were intraprocedural complications evaluated at the end of each LC concerning liver damage, gallbladder perforation, damage to cystic artery and duct, placement of the clip, and total operating time. Complications relating to damage to the liver were evaluated at the end of the training by reviewing the organ and assessing the injury of the gallbladder bed and surrounding liver parenchyma. The complication rate was evaluated using a 3-point Likert scale (supplementary material). Difficulty assessment of the ex vivo porcine liver was evaluated using the visual analog scale (VAS) on a scale ranging from 1 (extremely easy) to 10 (extremely hard) according to the given anatomical conditions such as organ size, aberrations of the cystic duct or artery, the thickness of the gallbladder wall or surrounding tissue [20]. Furthermore, successful completion of the LCs within 90 min and achieving the CVS were also assessed. LC was seen as successfully completed if there was no major damage to the surrounding structures of the gallbladder or liver, which could be a possible cause of intra or postoperative complications in a real-life scenario.

### Statistical analysis

Statistical analysis and descriptive statistics were performed using the SPSS software (version 25.0, IBM SPSS Inc., Chicago, Illinois, USA), and data are given as absolute frequency and mean ± standard deviation. Differences between the LC were assessed using the t-test for independent samples in parametric data and the Mann–Whitney U test for independent samples in the case of non-parametric data. For binary endpoints, group differences were calculated using the Chi-square test. Multivariable regression was performed to assess the influence of demographic parameters and personal characteristics on surgical performance. A *p* value of ≤ 0.05 was considered statistically significant.

## Results

All trainees completed the entire study (n = 40). During baseline LC, there was no statistically significant difference regarding the successful completion of the procedure and GOALS and OSATS (global and task-specific) scores between the two groups (Table 1). Regarding the total operating time, the iSurgeon group was significantly faster than the control group (90.5 ± 30.9 vs. 110.7 ± 22.2 min., *p* = 0.022).

**Table 1:** Parameters of baseline laparoscopic cholecystectomy

Parameter	iSurgeon group	Control group	*p* value
GOALS score global	13.1 ± 2.2	12.4 ± 1.9	0.322
GOALS score task-specific	4.4 ± 2.5	4.5 ± 1.4	0.938
OSATS score global	15.7 ± 3.0	15.8 ± 1.8	0.900
OSATS score task-specific	34.3 ± 10.0	31.6 ± 5.2	0.290
Total time (min)	90.5 ± 30.4	110.7 ± 22.2	0.022
Complication rate	4.6 ± 2.1	4.3 ± 2.1	0.653

Data are presented as number (percentage) for categorical variables, mean standard deviation ± for normally distributed or median, and [25th and 75th percentile] for not normally distributed continuous variables. Accordingly, Chi-Quadrat, exact Fisher, Student’s t-test, or Mann–Whitney U test was used to compare

*GOALS* global operative assessment of laparoscopic skills, *OSATS* Objective Structured Assessments of Technical Skills

### Primary endpoints

**Fig. 4:**
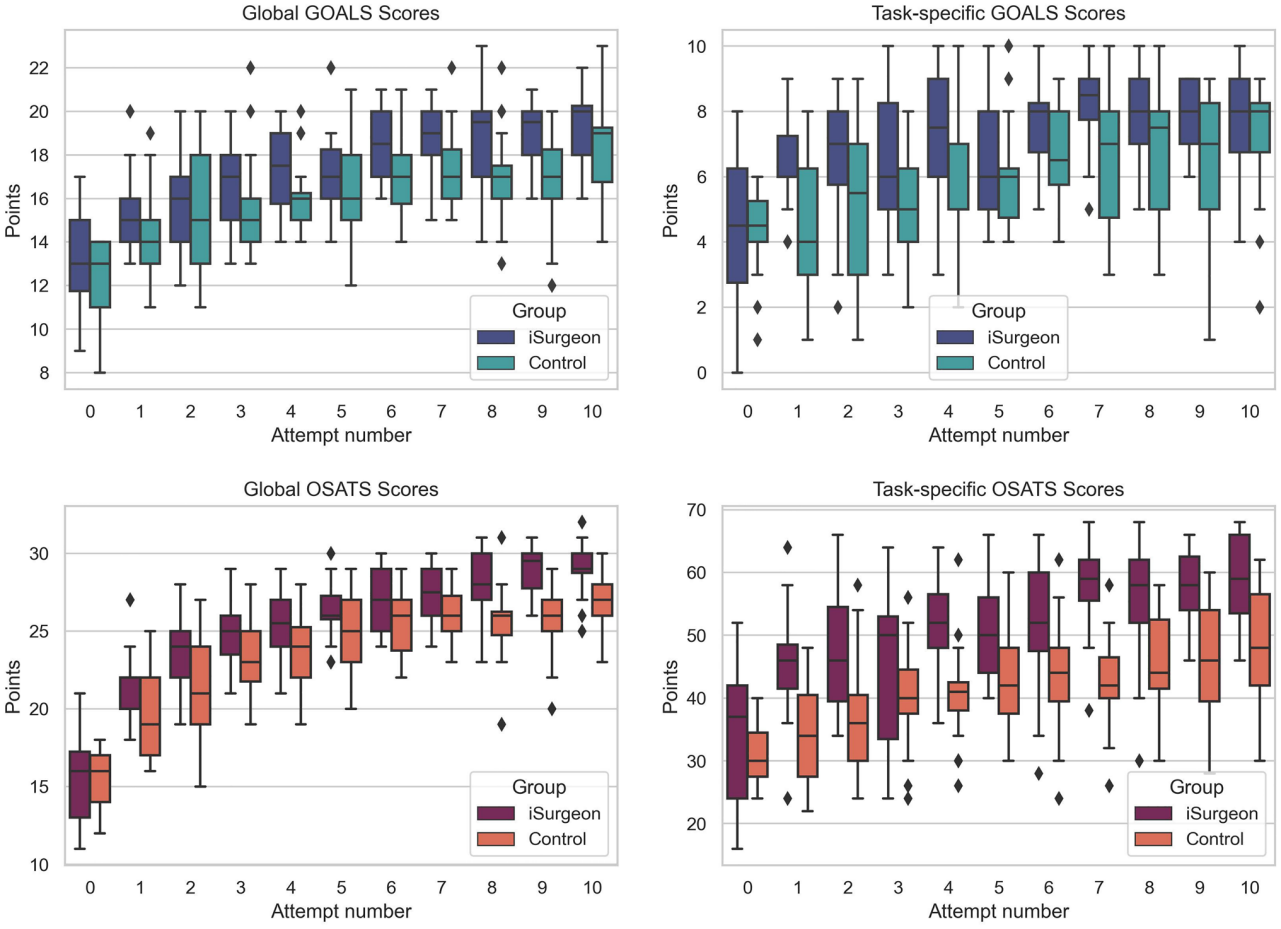
Comparison of global and task-specific GOALS and OSATS scores for all eleven LCs (0 is the baseline LC)

Training with the iSurgeon showed higher cumulative both global (25.3 ± 4.3 vs. 23.5 ± 4.0, *p* ≤ 0.001) and task-specific (50.8 ± 11.1 vs. 41.2 ± 9.4, *p* ≤ 0.001) OSATS scores. The cumulative global (17.3 ± 2.6 vs. 16.0 ± 2.6, *p* ≤ 0.001) and task-specific (6.99 ± 1.98 vs. 5.88 ± 2.09, *p* ≤ 0.001) GOALS scores were also higher in iSurgeon than in control group (Table 2). Guided by additional visual guidance through iSurgeon, the trainees within the iSurgeon group already showed better global (15.4 ± 1.7 vs. 14.1 ± 2.0, *p* = 0.030) and task-specific (6.5 ± 1.4 vs. 4.4 ± 23, *p* ≤ 0.001) GOALS, as well as global (21.8 ± 2.3 vs. 19.8 ± 2.7, *p* = 0.020) and task-specific (45.7 ± 8.6 vs. 34.3 ± 7.9, *p* ≤ 0.001) OSATS scores after performing first LC (Table 2). The tendency of performance improvement continued throughout all ten training sessions (Fig. 4).

**Table 2:** Comparison of postoperative parameters for all ten laparoscopic cholecystectomies

Parameter	iSurgeon group	Control group	*p* value
GOALS score global	17.3 ± 2.6	16.0 ± 2.6	≤ 0.001
GOALS score task-specific	7.0 ± 2.0	5.9 ± 2.1	≤ 0.001
OSATS score global	25.3 ± 4.3	23.5 ± 4.0	≤ 0.001
OSATS score task-specific	50.8 ± 11.1	41.2 ± 9.4	≤ 0.001
Total time (min)	79.6 ± 25.7	84.5 ± 33.2	0.087
Complication rate	2.7 ± 2.0	3.7 ± 2.0	≤ 0.001
CVS achievement (%)	79.5	41.4	≤ 0.001
Successful LC^a^ (%)	53.6	27.3	≤ 0.001
VAS score	51.5 ± 6.7	47.1 ± 8.4	≤ 0.001

Data are presented as number (percentage) for categorical variables, mean standard deviation ± for normally distributed or median, and [25th and 75th percentile] for not normally distributed continuous variables. Accordingly, Chi-Quadrat, exact Fisher, Student’s t-test, or Mann–Whitney U test was used to compare

*GOALS* global operative assessment of laparoscopic skills, *OSATS* Objective Structured Assessments of Technical Skills, *CVS* critical view of safety, *LC* laparoscopic cholecystectomy, *VAS* visual analog scale

^a^LC performed without major damage to the crucial anatomical structures within a time limit of 90 min

### Secondary endpoints

**Fig. 5:**
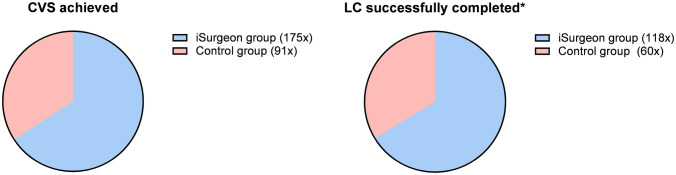
On the left: CVS achievement compared between the two groups. On the right: comparison of the LC’s successful completion within the 90 min time limit and without major damage to crucial anatomical structures

The secondary endpoints are presented in Table 2. The total operating time of all performed LCs did not differ between the iSurgeon and the control group (79.6 ± 25.7 vs. 84.5 ± 33.2 min, *p* = 0.087). The iSurgeon group had significantly fewer complications than the control group (2.7 ± 2.0 vs. 3.8 ± 2.0, *p* ≤ 0.001). Furthermore, successful completion of LCs within 90 min without major damage to the gallbladder, liver bed, or cystic duct and artery was performed in 53.6% in the iSurgeon group, while the control group showed only 27.3% successful completions (118 vs. 60 times, *p* ≤ 0.001). Additionally, the iSurgeon group achieved the CVS in 79.5% of the training sessions, while the control group achieved it in only 41.4% (175 vs. 91 times, *p* ≤ 0.001) (Fig. 5). According to the VAS, the difficulty of the LCs was higher in the iSurgeon group than in the control group (51.5 ± 6.7 vs. 47.1 ± 8.4, *p* ≤ 0.001).

## Discussion

Efficient training is still a challenge in the field of MIS. Several studies showed that telestration with AR showed great potential for improving surgical performance at the beginning of MIS training [5, 14, 21–26]. Studies using the iSurgeon have already demonstrated a positive impact on detecting the target structures and the performance of simple MIS tasks [14, 21].

There are several telestration devices with AR that have been developed to assist with surgical training and performance [5, 25, 27, 28]. One of them is a telestration device named PROXIMIE, and it allows remote experts to provide real-time, interactive guidance to on-site surgeons by drawing directly on a live video feed of the procedure [5, 27]. A similar telestration device was developed by Lacy et al., which is called AIS Telesurgeon [22]. These are just some of the telestration devices with AR that are, just like iSurgeon, developed to improve MIS performance and enhance MIS surgical training. However, telestration devices with AR still need to undergo further extensive trials of practical and clinical applications to prove their efficiency and benefits in a real-world setting.

In this randomized controlled study, the visual guidance with the iSurgeon contributed to significantly better performance and fewer complications in 10 repetitively performed LCs, regardless of the difficulty of the LCs, compared to the control group that had only verbal guidance. Assessment of the task-specific and global GOALS and OSATS scores showed significantly higher scores in the iSurgeon group for all LCs performed, proving that the use of the iSurgeon system with expert guidance improves MIS skills such as tissue and instrument handling, preparation, dissection as well as the flow of operation. Interestingly, this was already shown after the first LC was performed with the assistance of the iSurgeon. There are several studies that have reported a positive influence of telestration on performance [6, 14, 29]. Feng et al. developed a virtual pointing and telestration system utilizing the Microsoft Kinect movement sensor that facilitates the conveyance of expert knowledge by enabling trainers to point or draw on the laparoscopic screen [29]. The authors compared the effects of the telestration device on laparoscopic simulation training with conventional training. They saw no difference in time and error rates. However, they observed improved performance after the training with the telestration system compared to conventional training. Additionally, the economy of motion was significantly improved when using the telestration system, which indicates the improvement of the ability to use laparoscopic videos in more direct instrument handling [29]. The improved performance in medical students who were trained with iSurgeon was demonstrated through another study [6]. Wild et al. reported the superior intraoperative identification of intraoperative target structures during LC in medical students trained with iSurgeon compared to the ones receiving only verbal guidance during training. The medical students did not only perform better but also had less complications [6]. The reason for the observed skills improvement in the iSurgeon group could be the through real-time telestration with AR-enhanced communication between the tutor and the trainee. This communication enhancement during the surgical procedures could have positively influenced the performance of the iSurgeon group since communication issues can lead to mistakes and intraoperative complications [30, 31].

The transfer of this kind of MIS training to the OR is accompanied by potential risks of complications, especially during the learning phase of the exercising surgeon [11]. So far, evidence for a better performance using the iSurgeon was only demonstrated in ex vivo LCs. However, the visual guidance provided through iSurgeon may enable an optimized learning curve in MIS in a real-life setting, as LC is a commonly performed MIS procedure by young surgical residents in their early years of practice. Further studies with using the system need to be conducted to evaluate the effectiveness in a real-life scenario. There was no difference in total operating time between the two groups. Worth mentioning is that in previous studies conducted with the iSurgeon performing LC on the porcine liver, there was no evidence for improvement of operating time either [21]. A possible explanation for this is that all trainees were novices to LC, and usually, a certain number of procedures must be performed until competency is reached in MIS, allowing comparison [32]. Also, the visual explanation with iSurgeon might take some time in the beginning. However, Lopes et al. reported a positive impact of telestration with AR on time in medical students acquiring suturing skills [33]. Twenty students were randomized into telestration with AR and traditional on-site teaching groups. Both groups had obtained training in basic surgical suturing. The authors reported a faster global time in performing different sutures in telestration with the AR group compared to the control group. This could imply that the time improvement with telestration and AR is potentially more likely to be detected in acquiring basic surgical and procedural skills, whereas in more complex procedures this effect is less likely to be seen but the focus should rather be on improved guidance and training in complex operative situations and on avoidance of errors rather than time enhancement. It is substantial to emphasize that the quality of the procedure is more relevant than speed and improvement of operative time and, therefore, has inferior priority.

Interestingly, trainees in the iSurgeon group performed their baseline LC significantly faster than those in the control group. Nonetheless, this was only observed in the baseline LC. The two groups had no statistically significant difference in total operating time in the following 10 LCs.

Concerning safe execution of the procedures, the iSurgeon group showed more successful completion of LCs within 90 min compared to the control group. No major damage was induced on the liver, gallbladder, cystic duct, or artery, which could lead to potentially severe complications in a real-life scenario. These aspects of safe conduct during a surgical procedure correspond with findings that misunderstandings and lack of efficient communication can lead to intraoperative complications [31]. In their study, Wild et al. also observed fewer complications rate in the iSurgeon group compared to the control group, assigning this phenomenon to the improved communication between the tutor and the trainees, which arises from the visual component of the iSurgeon application, which was the focus of a study by Felinska et al. using eye-tracking technology [6, 14]. Feng et al. demonstrated the communication enhancement between the senior surgeons and the young surgical residents using the previously developed telestration system (Virtual Pointer system) in laparoscopic training, which led to subjectively better knowledge acquirement and less cognitive effort in learning laparoscopic skills for the trainees and the guidance for the tutor [34]. This supports the presented results of a telestration device with AR improving communication between the tutor and the trainee.

Additionally, according to the VAS, the iSurgeon group had a significantly lower complication rate than the control group despite more challenging LCs. This demonstrates how additional iSurgeon visual guidance potentially assists trainees to perform the LCs safely. It is comprehensible that the use of the iSurgeon with visual expert guidance assisted the trainees in developing a better understanding of the anatomical structures and procedure itself, leading to a more effortless flow from one procedural move to the next independently and being able to perform the procedure more often within the given time limit.

Furthermore, visual guidance helped the trainees in the iSurgeon group achieve the CVS more often, which is one of the crucial steps in LC and essential in preventing damage to the surrounding structures [35]. This is essential to minimize bile duct injuries that can cause life-altering complications and significant morbidity [36–38].

Finally, the iSurgeon group had more difficult gallbladders to work with, assessed using the VAS before each training. Considering the lower rate of complications and a higher percentage of successfully performed LCs, the iSurgeon group benefitted from visual guidance even in difficult cases, making the iSurgeon a potentially valuable tool in preclinical and clinical scenarios. Telestration devices with AR, like the iSurgeon, could have an additional effect on the learning curves of surgical trainees and patients' safety. Also, the iSurgeon could show potential in training young aspiring surgeons when used in specialized training centers or upon transfer to the OR with proctoring by a senior surgeon.

However, despite promising opportunities that iSurgeon offers in MIS training, its routine application in everyday MIS training is still not accessible to all institutions due to its ongoing development. To implement the iSurgeon in training centers or the OR, it is necessary to have the corresponding material and software, which could potentially make it difficult to implement such technology without the required financial support. Nevertheless, the benefit of such assistance systems in MIS training should encourage institutions to invest in further research to make such technology accessible to improve surgical performance and quality and, through that, hopefully, patient outcomes.

### Limitations

Due to the nature of the study and the spatial and temporal conditions, trainees from the iSurgeon and control group occasionally worked together in the same room. Thus, the flow of information between the two groups could have been a potential bias in both directions and within the two groups. Furthermore, since the training was conducted with students in different years of medical school (3rd to 6th year), a potential bias concerning the different levels of surgical education might have occurred. The inclusion criterium was the completion of the first module of the complementary MIS training curriculum, before which none of the participants had had any laparoscopic experience, but further previous surgical education was not assessed. However, besides the total time, the performance scores were comparable in the baseline LC. It is also important to mention that the intervention group had performed the baseline LC faster compared to the control group. This puts potential bias in the randomization process. However, the performance scores and all other compared parameters of the baseline LC were comparable between the two groups. Therefore, the study proceeded according to the predefined study protocol. One of the further limitations of the study is its small sample size, which could potentially influence the outcomes of the study.

Finally, one must say that the environment in the training center where the LCs on porcine livers have been performed is not entirely comparable to an OR with a sterile covered operating field and that sound levels and disruptive factors due to the operating team consisting of surgeons, anesthesiologist, and nursing staff are higher in a real-life scenario. Nevertheless, this is also a chance for implementing the iSurgeon in a real-life OR setting to allow guidance when verbal feedback is difficult. This might, therefore, result in even higher benefits from telestration with AR in real-life conditions.

## Conclusion

MIS training with the iSurgeon telestration system with AR showed better task-specific and overall MIS performance for the first ten repeated ex vivo LCs. Trainees with verbal and visual guidance completed more LCs within the time limit and performed fewer complications despite higher difficulty levels of the LCs compared to those receiving only verbal guidance. This study underlines the value of visual guidance using telestration with AR in MIS training. Further studies should assess the benefits of incorporating the iSurgeon clinical training on patients.

### Supplementary Information

Below is the link to the electronic supplementary material.

Supplementary file1 (DOCX 96 kb)
