# Supplementary figures and images for: Telestration with augmented reality improves the performance of the first ten ex vivo porcine laparoscopic cholecystectomies: a randomized controlled study
Source: Surg Endosc. 2023 Aug 23;37(10):7839–48. doi: 10.1007/s00464-023-10360-y (PMC10520207; doi:10.1007/s00464-023-10360-y)

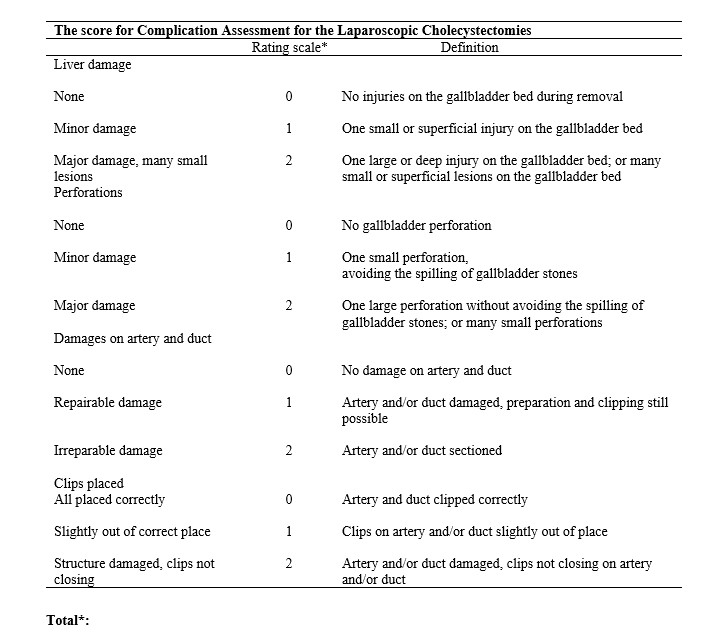

Supplement: Supplementary file 1 — Supplementary file1 (DOCX 96 kb) [file 464_2023_10360_MOESM1_ESM.docx]
